# The District Nurse

**Published:** 1924-08

**Authors:** M. Ida Batty


### CORRESPONDENCE.

[Correspondence on all subjects is invited, but we cannot be responsible for the opinions expressed by our correspondents who must give name and address as a guarantee of good faith, but not necessarily for publication. Correspondents are reminded that conciseness greatly facilitates early insertion.]

### The District Nurse.

*To the Editor of* THE HOSPITAL AND HEALTH REVIEW.

Sir,—In the address on the training of nurses reported in your July issue, Sir Wilmot Herringham asks why educated ladies do not enter the nursing profession as they did when he was young ?

I should like to give a few reasons why I think they abstain from one particular branch of the profession—District Nursing—especially in rural areas. I should also like to mention one or two ways in which I think this most important and necessary branch could be made more attractive. The local Nursing Committees often show very little consideration to the nurse with regard to her domestic and physical comfort. She is given a cottage to live in, sometimes of the most primitive type, with neither water laid on nor proper sanitary arrangements, and certainly no bath. She has little comfort in the house, for owing to her small salary she is unable to employ any efficient help. Her meals are, therefore, indifferently prepared. She is given scarcely any social standing in the village, consequently her life, apart from her work, is a very lonely one. The arrangements made for her free week-ends are vague.

I consider that these short holidays should be quite definitely fixed and rigorously adhered to. The method of payment in many instances is not a good one. Sometimes she is paid a salary of £50 or £60 a year, but boarded and housed by a local cottager whom the Committee pay. Her rooms are, therefore, small and the furnishing often crude and depressing, with little space for any of her own personal belongings. Occasionally the nurse is paid a small salary and given a house free of rent. This rent may either be so little that added to her salary it may only mean the addition of another £10, or she may be given a Council house to live in, the rental of which may amount to £40 per annum. If her salary is £150, it will readily be seen that about a quarter of her whole income is taken up in rent.

The following are a few ways in which I think the work might be made more attractive. The Secretary of the Nursing Association should be a lady with a wide sympathy and deeply interested in the work, so that she will make herself thoroughly conversant with the difficulties that the nurse has to contend with, and realise that the lady undertaking district nursing most certainly is a nurse, but that she is also a woman of ability and education, with interests and desires outside her calling. She should have the opportunity of choosing, as far as the present-day housing difficulty will allow, accommodation suited to her taste, and not be forced to take whatever the Committee provides. A relief nurse should be engaged at specified intervals irrespective of the number of cases on the books at the time. If there is no suitable person in the neighbourhood who could discharge the temporary duties or the local doctor is too busy to undertake an emergency, a nurse can always be obtained at a moment's notice from any of the large co-operations. She should be paid not only a bare living wage but a salary in keeping with her skill and long and arduous apprenticeship.

The Committee's lack of money is often due to defective organisation in collecting. The poorest people can nearly all afford a few pence a week, but are quite unable to pay a few shillings once a quarter. In these instances it would therefore be necessary to institute a system of weekly collecting Those people who are in a secure position financially could pay either quarterly or half-yearly. Should the services of the nurse be required by the more leisured classes, I would suggest a fee of from 5s. to 10s. per visit being paid into the Association. The annual Bank Holiday fêtes held in many rural districts sometimes make a profit of £200. Surely the Committee could be induced to set aside a substantial portion of this for expenses in connection with the Nursing Association.

I feel deeply that District Nursing should not be entrusted to partially-trained women, and I confidently anticipate the time when, the drawbacks which I have pointed out being overcome and the prevailing conditions ameliorated, well-educated and highly-qualified women will be induced to take up this branch of work, in order that our sick and aged poor may receive the very best attention possible.

M. IDA BATTY.

